# Effects of Length and Loop Composition on Structural Diversity and Similarity of (G_3_TG_3_N_m_G_3_TG_3_) G-Quadruplexes

**DOI:** 10.3390/molecules25081779

**Published:** 2020-04-13

**Authors:** Jie Li, I-Te Chu, Ting-An Yeh, De-Yu Chen, Chiung-Lin Wang, Ta-Chau Chang

**Affiliations:** Institute of Atomic and Molecular Sciences, Academia Sinica, Taipei 106, Taiwan; julielee0531@gmail.com (J.L.); r04223204@gmail.com (I.-T.C.); tati31233@gmail.com (T.-A.Y.); ymonkeyy@gmail.com (D.-Y.C.); q102081@gmail.com (C.-L.W.)

**Keywords:** G-quadruplex, structural diversity, structural similarity, loop length, base composition, flanking nucleotide, quadruplex-duplex junction

## Abstract

A G-rich sequence containing three loops to connect four G-tracts with each ≥2 guanines can possibly form G-quadruplex structures. Given that all G-quadruplex structures comprise the stacking of G-quartets, the loop sequence plays a major role on their folding topology and thermal stability. Here circular dichroism, NMR, and PAGE are used to study the effect of loop length and base composition in the middle loop, and a single base difference in loop 1 and 3 on G-quadruplex formation of (G_3_HG_3_N_m_G_3_HG_3_) sequences with and without flanking nucleotides, where H is T, A, or C and N is T, A, C, or G. In addition, melting curve for G-quadruplex unfolding was used to provide relatively thermal stability of G-quadruplex structure after the addition of K^+^ overnight. We further studied the effects of K^+^ concentration on their stability and found structural changes in several sequences. Such (G_3_HG_3_N_m_G_3_HG_3_) configuration can be found in a number of native DNA sequences. The study of structural diversity and similarity from these sequences may allow us to establish the correlation between model sequences and native sequences. Moreover, several sequences upon interaction with a G-quadruplex ligand, BMVC, show similar spectral change, implying that structural similarity is crucial for drug development.

## 1. Introduction

A large number of guanine (G)-rich sequences are found in the human genome [1,2,3]. In general, G-rich sequences may adopt into various G-quadruplex (G4) structures, which depend on their sequences and lengths, loop composition, and flanking nucleotides in K^+^ solution [4,5,6,7,8,9,10,11,12]. Accumulating evidence on the biological functions of G4 structures in regulating multiple cellular processes [13,14,15,16,17,18] and imaging-based studies on G4 antibodies and G4 ligands for visualizing the presence of G4s in cells [19,20,21,22,23] support the existence of G4 structure in vivo. Given that structural properties of G4s are critical for biomedical implication and drug development, many questions on structural variation as a result of sequence deviation are yet to be addressed. For example, under what sequence variations, one could infer rules for predicting structural similarity in G4s. 

Previously, we found the structural diversity of a mitochondrial sequence mt10251 (G_3_TG_3_AGTAGTTCCCTGCTAAG_3_AG_3_), including the coexistence of a hairpin structure and monomeric, dimeric, and tetrameric G4 structures in 20 mM K^+^ solution. Moreover, a single-base mutation of mt10251 could cause significant changes in terms of structural populations and polymorphism [24]. Particularly, most single-base mutated sequences of mt10251 favor dimeric G4 formation in the absence of hairpin formation. This sequence contains four G-tracts with three consecutive G-bases without flanking nucleotides separated by three loops with 1, 16, and 1 nt (1,16,1 loop lengths). We anticipate that the dimeric G4 formation is mainly due to the sequences of G_3_TG_3_ and G_3_AG_3_ at two ends. It is of interest to examine whether the change of the middle loop length of (G_3_HG_3_N_m_G_3_HG_3_) could also favor dimeric G4 formation, where H = A, T, or C and N = A, T, C, or G.

The G-rich (G_3_HG_3_N_m_G_3_HG_3_) configuration can be found in a number of native DNA sequences. A G-rich sequence Pu22 (TGAG_3_TG_3_TAG_3_TG_3_TAA) in the c-MYC promoter region adopts into a monomeric parallel G4 structure [25,26]. The deletion of 5′-TGA and 3′-TAA from Pu22 satisfies the category of (G_3_HG_3_N_m_G_3_HG_3_). In addition, Kuryavyi and Patel reported that a G-rich human *chl1* intronic sequence (G_3_TG_3_GAAGG_3_TG_3_T) also adopts a monomeric G4 structures in K^+^ solution [27]. Lim et al. [28] demonstrated that a G-rich sequence GTERT (AGG_3_AG_3_GCTG_3_AG_3_C) in the hTERT promoter can form two monomeric G4 structures. Similarly, Onel et al. [29] found that a G-rich sequence PIG4 (CG_3_CG_3_AGCGCGGCGGGCG_3_CG_3_CA) located at the upstream of the human BCL2 gene P1 promoter can form two different monomeric G4 structures containing a stem-loop hairpin. These findings suggest that the addition of flanking nucleotides to the G-rich sequence of (G_3_HG_3_N_m_G_3_HG_3_) prefer to form monomeric G4 structure. It is curious to examine whether the deletion of the flanking nucleotides in these G-rich sequences will favor the dimeric G4 formation. 

**Figure 1:**
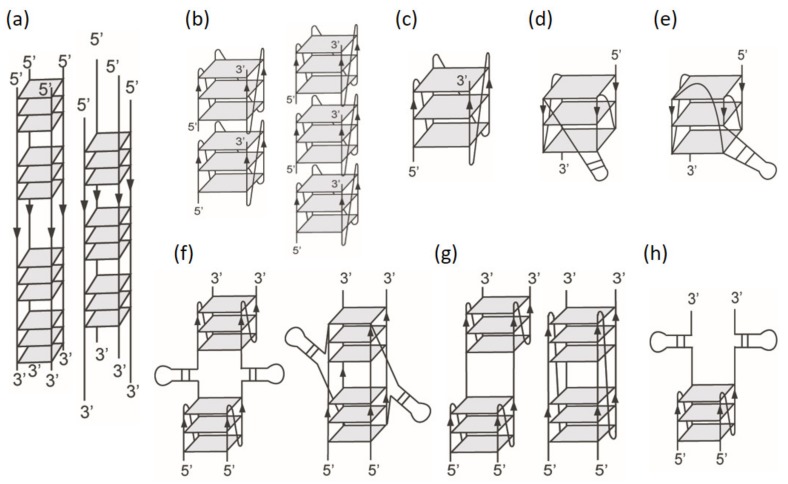
Proposed G4 topologies in this study. (**a**) parallel tetramers, (**b**) stacking of parallel monomers, (**c**) parallel monomer, (**d**) parallel quadruplex-duplex monomer, (**e**) unprecedented quadruplex-duplex monomer, (**f**) parallel quadruplex-duplex dimers, (**g**) parallel quadruplex dimers, (**h**) quadruplex-duplex dimer with one quadruplex.

In this work, we use nuclear magnetic resonance (NMR) for identifying G4 formation, circular dichroism (CD) for distinguishing parallel and nonparallel G4 structures, and polyacrylamide gel electrophoresis (PAGE) for classifying monomeric, dimeric, and tetrameric G4 formation of a number of G-rich sequences in K^+^ solution. Particularly, imino proton NMR spectra can provide distinct peaks near 13 ppm for duplex signals of G-base, near 13.5 ppm for duplex signals of T-base, and 10−12.5 ppm for G4 signals [30]. Such imino proton NMR signals are important in the comparison of structural diversity and similarity of these G-rich sequences. Since the 100 μM DNA concentration is normally used to obtain imino proton NMR spectra, this DNA concentration is applied for all the experiments in this work. We first examine the loop effect, particularly, on the formation of dimeric G4 structure by changing the middle loop size in the G-rich sequence of (G_3_HG_3_N_m_G_3_HG_3_). For simplicity, we tentatively label each sequence by using the characters of the three loops, such as T16T for G_3_TG_3_TTGCGCAATTGCGCTTG_3_TG_3_. Considering the similarity between (G_3_TG_3_TAG_3_TG_3_) and (TGAG_3_TG_3_TAG_3_TG_3_TAA), we further study the (G_3_HG_3_N_m_G_3_HG_3_) sequences with the addition of flanking nucleotides (FN) at both ends of 5′-TGA and TAA-3′ to examine whether they form monomeric G4 structures. The sequences studied in this work are listed in Table 1. In addition, CD melting is used to define quadruplex stability and G4 binding ligand is applied to examine structural diversity and similarity. Since such G-rich sequences (G_3_HG_3_N_m_G_3_HG_3_) are found in a number of native DNA, it is important to explore if there are some common features of their G4 structures with and without the flanking nucleotides. Figure 1 shows the proposed G4 topologies in this work.

## 2. Results

### 2.1. Effect of Loop Length on G4 Formation of (G_3_TG_3_N_m_G_3_TG_3_) Sequences

**Figure 2:**
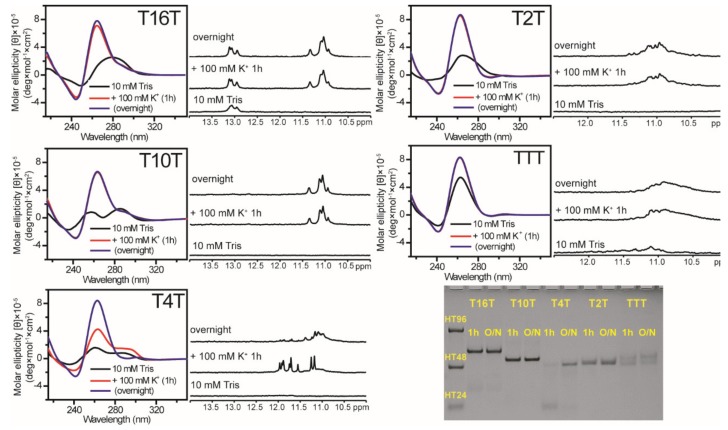
The effect of loop length on G4 formation of (G_3_TG_3_N_m_G_3_TG_3_) sequences. CD and NMR spectra of T16T, T10T, T4T, T2T, and TTT in 10 mM Tris and after 1 h and overnight (O/N) addition of 100 mM K^+^ together with their PAGE assays of marker bands of HT24 (T_2_AG_3_)_4_, HT48 (T_2_AG_3_)_8_, and HT96 (T_2_AG_3_)_16_ (lane 1) and each sequence after 1 h and O/N addition of 100 mM K^+^ for T16T, T10T, T4T, T2T, and TTT. The same DNA concentration of 100 μM was used in the experiments of CD, NMR and PAGE of this work.

The CD spectra of T16T, T10T, T2T, and TTT sequences showed similar intensity at a major 265 nm band after 1 h and overnight addition of 100 mM K^+^, implying that the growth of parallel G4 structure is predominant for these G-rich sequences (Figure 2). NMR spectra of each sequence were also similar after 1 h and overnight addition of 100 mM K^+^. Particularly, similar spectral patterns were detected in the region of 10−12 ppm between T16T and T10T (Figure 2). It is likely that T16T and T10T form similar type of G4 structure, except the coexistence of duplex formation in T16T based on the presence of imino proton signals near 13 ppm. The detection of imino proton NMR signals in the region of 12.5−14.0 ppm is due to Watson-Crick (WC) hydrogen bond. Of interest was that the CD spectra of T4T showed spectral change from a major 265 nm band together with a minor 290 nm band to a single 265 nm band after the addition of 100 mM K^+^ from 1 h to overnight (Figure 2). Spectral change was also detected in its imino proton NMR spectra. Of interest is that the PAGE results of T16T, T10T, T2T, and TTT sequences showed a predominant band of multimeric G4 formation after 1 h and overnight addition of 100 mM K^+^, while the PAGE assays of T4T showed the change from a band of monomeric G4 formation to a major band of multimeric G4 formation after the addition of 100 mM K^+^ from 1 h to overnight. Similar results on these sequences were also observed after the addition of 20 mM K^+^ (data not shown). In this work, the same 100 μM DNA concentration was used in CD, NMR, and PAGE experiments. Our findings suggested that these (G_3_TG_3_N_m_G_3_TG_3_) sequences without flanking nucleotides favor multimeric G4 formation. Notably, the similar gel migrations of TTT (15-mer) and T10T (24-mer) in PAGE suggested that T10T is likely due to a dimer, while TTT is probably due to a trimer or even a tetramer. 

### 2.2. Effect of Base Composition in the Middle Loop on G4 Formation of (G_3_TG_3_N_4_G_3_TG_3_) Sequences

**Figure 3:**
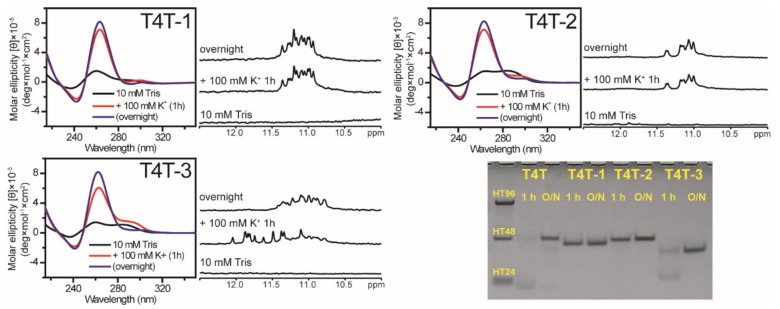
The effect of middle loop base on G4 formation of (G_3_TG_3_N_4_G_3_TG_3_) sequences. CD and NMR spectra of T4T-1, T4T-2, and T4T-3 in 10 mM Tris and after 1 h and overnight (O/N) addition of 100 mM K^+^ together with their PAGE assays of marker bands of HT24 (T_2_AG_3_)_4_, HT48 (T_2_AG_3_)_8_, and HT96 (T_2_AG_3_)_16_ (lane 1) and each sequence after 1 h and O/N addition of 100 mM K^+^ for T4T, T4T-1, T4T-2, and T4T-3. The same DNA concentration of 100 μM was used in the experiments of CD, NMR and PAGE of this work.

Here the spectral change from a monomeric G4 structure to a multimeric G4 structure was clearly detected in T4T after the addition of 100 mM K^+^, while the monomeric G4 structure was negligible for T16T, T10T, T2T, and TTT sequences. To examine whether such structural conversion can be affected by the change of middle loop sequence of T4T, we studied three more sequences by changing the middle loop bases, ATTA for T4T-1, CATC for T4T-2, and ACGT for T4T-3. Their CD and NMR results showed that the spectral change was also detected in T4T-3, but not found in T4T-1 and T4T-2 after the addition of 100 mM K^+^ (Figure 3). In addition, the PAGE assays of T4T-3 supported the structural change from monomeric G4 formation to multimeric G4 formation after the addition of 100 mM K^+^ from 1 h to overnight. However, the details of structural conversion detected in T4T and T4T-3, but not in T4T-1 and T4T-2 deserve further study. Here their imino proton NMR spectra showed different spectral patterns, implying that their folding patterns depend upon base composition in the middle loop. 

### 2.3. Effect of Flanking Nucleotides on G4 Formation of (G_3_TG_3_N_m_G_3_TG_3_)-FN Sequences

**Figure 4:**
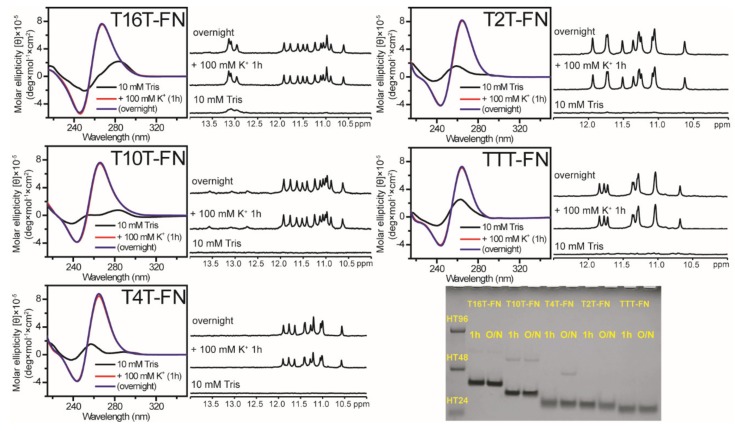
The effect of flanking nucleotides on G4 formation of (G_3_TG_3_N_m_G_3_TG_3_)-FN sequences. CD and NMR spectra of T16T-FN, T10T-FN, T4T-FN, T2T-FN, and TTT-FN in 10 mM Tris and after 1 h and overnight (O/N) addition of 100 mM K^+^ together with their PAGE assays of marker bands of HT24 (T_2_AG_3_)_4_, HT48 (T_2_AG_3_)_8_, and HT96 (T_2_AG_3_)_16_ and each sequence after 1 h and O/N addition of 100 mM K^+^ for T16T-FN, T10T-FN, T4T-FN, T2T-FN, and TTT-FN. The same DNA concentration of 100 μM was used in the experiments of CD, NMR and PAGE of this work.

Given that Pu22 forms monomeric parallel G4 structure in K^+^ solution, we further studied the above G-rich sequences with the addition of flanking nucleotides (FN) at both ends of 5′-TGA and TAA-3′ (Table 1, Figure 4). Their CD spectra showed a positive band near 265 nm together with a negative band near 245 nm after the addition of 100 mM K^+^, suggesting that they all form parallel G4 structures. The NMR spectra of T16T-FN and T10T-FN showed almost identical imino proton signals in the region of 10−12.5 ppm, which differ from similar spectral patterns detected in T4T-FN and T2T-FN. However, the imino proton signals detected in the region of 12.5–14.0 ppm in T10T-FN were very different from that in T16T-FN. Of interest was the fact that the imino proton signals detected in the region of 12.5−14.0 ppm in T10T-FN are not detected in T10T after the addition of 100 mM K^+^. The PAGE results showed a predominantly monomeric band for all of them. According to the gel migration, a weak dimeric band was detected in the gel migration of T10T-FN (30-mer) and T4T-FN (24-mer) in 100 mM K^+^ solution. Notably, such dimeric band was negligible in the gel migration in 20 mM K^+^ solution. In addition, the imino proton signals in the region of 12.5–14.0 ppm in T10T-FN were not detected in 20 mM K^+^ solution (data not shown). Our results illustrated that these (G_3_TG_3_N_m_G_3_TG_3_)-FN sequences form predominantly monomeric G4 structures even after the addition of 100 mM K^+^. Furthermore, time-dependent CD studies showed that the growth of 265 nm CD signal of monomeric G4 formation for (G_3_TG_3_N_m_G_3_TG_3_)-FN sequences is more rapid than that of multimeric G4 formation for (G_3_TG_3_N_m_G_3_TG_3_) sequences after the addition of 20 mM K^+^ (Figure S1).

### 2.4. Effect of a Single Base Difference in Loop 1 and 3 on G4 Formation of (G_3_HG_3_N_m_G_3_HG_3_)-FN Sequences

Given that T16T and T10T sequences showed almost identical CD and NMR spectral patterns, we further examined how a single base modification in loop 1 and 3 can affect the G4 structure. The PAGE results of A16T, A10A, and C10C sequences showed a predominant band of dimeric formation after the addition of 100 mM K^+^ (Figure S2). Of interest was that the CD and NMR spectra of these sequences showed almost identical spectral patterns in the 10−12.5 ppm region, suggesting that such change has no appreciable effect on their G4 structures. Similar results were also observed after the addition of 20 mM K^+^ (data not shown). 

**Figure 5:**
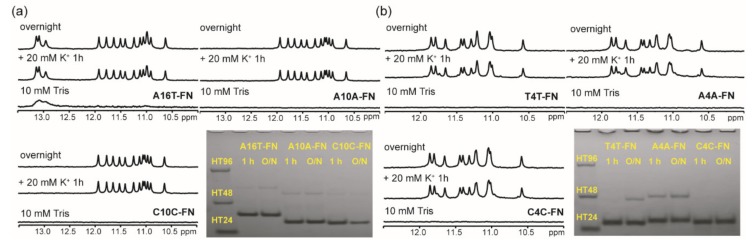
The effect of a single base change in loop 1 and 3 on G4 formation of (G_3_HG_3_N_m_G_3_HG_3_)-FN sequences. (**a**) NMR spectra of A16T-FN, A10A-FN, and C10C-FN in 10 mM Tris and after 1 h and overnight (O/N) addition of 20 mM K^+^ together with their PAGE assays of marker bands of HT24 (T_2_AG_3_)_4_, HT48 (T_2_AG_3_)_8_, and HT96 (T_2_AG_3_)_16_ and each sequence after 1h and O/N addition of 20 mM K^+^ for A16T-FN, A10A-FN, and C10C-FN. (**b**) NMR spectra of T4T-FN, A4A-FN, and C4C-FN in 10 mM Tris and after 1 h and O/N addition of 20 mM K^+^ together with their PAGE assays of marker bands of HT24 (T_2_AG_3_)_4_, HT48 (T_2_AG_3_)_8_, and HT96 (T_2_AG_3_)_16_ and each sequence after 1h and O/N addition of 20 mM K^+^ for T4T-FN, A4A-FN, and C4C-FN. The same DNA concentration of 100 μM was used in the experiments of NMR and PAGE of this work.

The imino proton NMR spectra of A16T-FN, A10A-FN, and C10C-FN showed almost identical signals in the region of 10−12.5 ppm in 20 mM K^+^ solution (Figure 5a) and 100 mM K^+^ solution (Figure S3). However, the imino proton signals in the region of 12.5−14.0 ppm of A10A-FN and C10C-FN were also detected in 100 mM K^+^ solution, but not detected in 20 mM K^+^ solution. The CD spectra suggested that they all form parallel type of G4 structures (data not shown). Of interest was the fact that the PAGE results of A10A-FN and C10C-FN showed a predominantly monomeric band in 20 mM K^+^ solution (Figure 5a), but a marked increase in dimeric population in 100 mM K^+^ solution (Figure S3). We found no appreciable change in the imino proton NMR signals in the 10.0−12.5 ppm region of A10A-FN and C10C-FN, even though there is marked increase in dimeric population in 100 mM K^+^ solution. Nevertheless, our results suggested that a single-base difference of A, T, and C in loop 1 and loop 3 of H10H-FN sequences makes no appreciable change on their monomeric G4 structures.

In addition, the imino proton NMR spectra of T4T-FN, A4A-FN, and C4C-FN also showed almost identical signals in the region of 10−12.5 ppm in 20 mM K^+^ solution (Figure 5b). The PAGE results supported that they favor to form monomeric G4 structure. The CD spectra suggested that they all form parallel type of G4 structure (data not shown). In addition, their NMR spectral patterns were also similar after 1 h addition of 100 mM K^+^. However, the imino proton NMR signals of A4A-FN showed some difference from that of T4T-FN and C4C-FN after overnight addition of 100 mM K^+^ (Figure S4). Of interest was that the PAGE assays of A4A-FN showed a large change from monomeric population to dimeric population as a function of time in 100 mM K^+^ solution. It is not clear why such change is less detected in T4T-FN and not detected in C4C-FN. Here the imino proton NMR spectra also suggested that a single-base difference of A, T, and C in loop 1 and loop 3 in (G_3_HG_3_N_4_G_3_HG_3_)-FN sequences has no appreciable effect on their monomeric G4 structures. 

### 2.5. Effect on G4 Formation of Native G-Rich Sequences without Flanking Nucleotides

**Figure 6:**
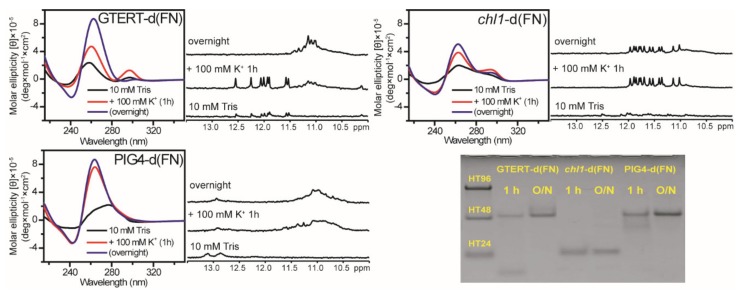
The Effect on G4 formation of native G-rich sequences without flanking nucleotides. CD and NMR spectra of GTERT-d(FN), PIG4-d(FN), and *chl1*-d(FN) in 10 mM Tris and after 1 h and overnight (O/N) addition of 100 mM K^+^ together with their PAGE assays of marker bands of HT24 (T_2_AG_3_)_4_, HT48 (T_2_AG_3_)_8_, and HT96 (T_2_AG_3_)_16_ and each sequence after 1 h and O/N addition of 100 mM K^+^ for GTERT-d(FN), *chl1*-d(FN), and PIG4-d(FN). The same DNA concentration of 100 μM was used in the experiments of CD, NMR and PAGE of this work.

In addition to Pu22, a number of native G-rich sequences containing (G_3_HG_3_N_m_G_3_HG_3_) sequences with flanking nucleotides, such as *chl1* [27], GTERT [28], and PIG4 [29] were previously studied by NMR, and they all favored to form monomeric G4 structures. Here we examined whether these sequences without flanking nucleotides favor to form multimeric G4 structures. The CD spectra showed a major growth of the 265 nm band together with a minor growth of the 295 nm band for *chl1*-d(FN) and GTERT-d(FN) and a growth of the 265 nm band for PIG4-d(FN) after 1 h addition of 100 mM K^+^. After overnight addition of 100 mM K^+^, the 265 nm band kept growth, while the 295 nm band decreased for *chl1*-d(FN) and GTERT-d(FN) (Figure 6). In addition, NMR spectra showed fine imino proton signals for *chl1*-d(FN), spectral change from fine signals to broad band for GTERT-d(FN), and broad band for PIG4-d(FN) after the addition of 100 mM K^+^ (Figure 6). In addition, PAGE assays showed a predominantly monomeric band for *chl1*-d(FN), conformational change from a monomeric band to a multimeric band for GTERT-d(FN) (17-mer), and a major dimeric band for PIG4-d(FN) (26-mer) after the addition of 100 mM K^+^ (Figure 6). These findings indicated that PIG4-d(FN) and GTERT-d(FN) also favor to form multimeric G4 structures. However, *chl1*-d(FN) forms monomeric G4 structure after the addition of 100 mM K^+^.

The question is why *chl1*-d(FN) does not favor multimeric G4 formation after the addition of 100 mM K^+^. This is probably due to four consecutive G bases in the second and third G-tracts of *chl1*-d(FN) because the flexibility of four consecutive G bases can change a single T-base in loop 1 and in loop 3 as (G_3_TG_3_GAAGG_3_TG_3_) to a TG in loop 1 and a GT in loop 3 as (G_3_TGG_3_AAG_3_GTG_3_). We further studied CD, NMR, and PAGE of T_2_4T_2_ (G_3_TTG_3_CATGG_3_TTG_3_) and T_2_4T (G_3_TTG_3_CATGG_3_TG_3_). The results showed that these two sequences formed monomeric G4 structures instead of multimeric G4 structures after the addition of 100 mM K^+^ (Figure S5). Thus, it is likely that G-rich sequences containing (G_3_HG_3_N_m_G_3_HG_3_) without four consecutive G-bases favor multimeric G4 formation.

### 2.6. Effect of Flanking Nucleotides on G4 Formation of mt10251-FN (mt10248)

**Figure 7:**
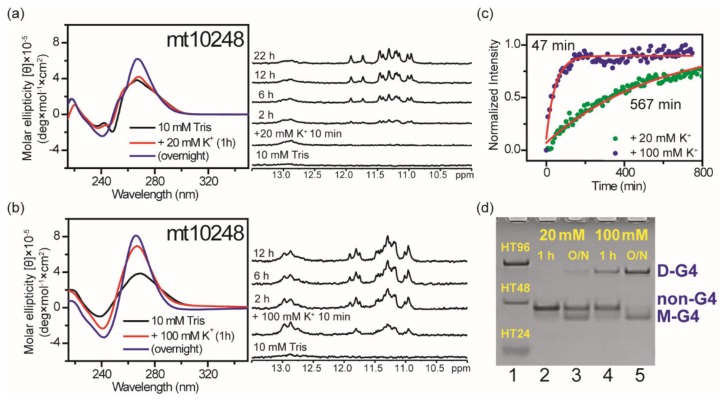
The [K^+^] dependent study on the effect of flanking nucleotides on G4 formation of mt10251-FN (mt10248). Time-dependent CD and NMR spectra of mt10248 in 10 mM Tris and after the addition of 20 mM K^+^ (**a**) and 100 mM K^+^ (**b**). The plots of arising CD signals at 265 nm of mt10248 fitted with single exponential after the addition of 20 mM K^+^ and 100 mM K^+^ (**c**). PAGE assays of marker bands of HT24 (T_2_AG_3_)_4_, HT48 (T_2_AG_3_)_8_, and HT96 (T_2_AG_3_)_16_ and mt10248 after 1h and O/N addition of 20 mM K^+^ and 100 mM K^+^ (**d**). The same DNA concentration of 100 μM was used in the experiments of CD, NMR and PAGE of this work.

Given that the third G-tract of G24−G26 may be involved in hairpin formation of mt10251 in 10 mM Tris [24], we examined whether a native sequence, CCAG_3_TG_3_AGTAGTTCCCTG CTAAG_3_AG_3_TAG (mt10248), which simply adds native flanking nucleotides to the mt10251 sequence with 5′-CCA and TAG-3′ at two ends, could form a monomeric G4 structure. The NMR spectra of mt10248 showed very weak signals near 13 ppm in 10 mM Tris, implying that hairpin formation is almost negligible. However, the imino proton signals near 13 ppm was detected right after the addition of 20 mM K^+^ and then decreased slowly, whereas imino proton signals in the region of 10−12.5 ppm were detected later and increased slowly (Figure 7a). Of interest is that both signals near 13 ppm and in the region of 10−12.5 ppm were detected right after the addition of 100 mM K^+^ (Figure 7b). The increase of positive CD bands at 265 nm is a typical CD pattern for parallel G4 structures. In addition, CD spectra showed that the increase of the 265 nm band intensity is very slow after the addition of 20 mM K^+^ and relatively faster after the addition of 100 mM K^+^ (Figure 7a,b). Further study of time-dependent CD signal at 265 nm showed that the arising time about 570 min after the addition of 20 mM K^+^ is much longer than the arising time about 47 min after the addition of 100 mM K^+^ (Figure 7c). PAGE results suggested that only small portion of mt10248 formed monomeric G4 structure after the addition of 20 mM K^+^ overnight. Of importance is the fact that a small portion of mt10248 remains in monomeric G4 structure, while most of mt10248 forms a major component of dimeric G4 structure after the addition of 100 mM K^+^ overnight (Figure 7d). The studies of mt10248 illustrated that low K^+^ concentrations favor monomeric G4 formation, while high K^+^ concentrations favor dimeric G4 formation. These findings demonstrated the effect of K^+^ concentration that could cause marked changes in terms of structural populations and polymorphism. Although a G-tract in mt10248 could be involved in hairpin formation, its monomeric G4 structure was co-existed and detected after the addition of K^+^. 

### 2.7. Effect of K^+^ Concentration on Thermal Stability of (G_3_HG_3_N_m_G_3_HG_3_) G4 Structures

**Figure 8:**
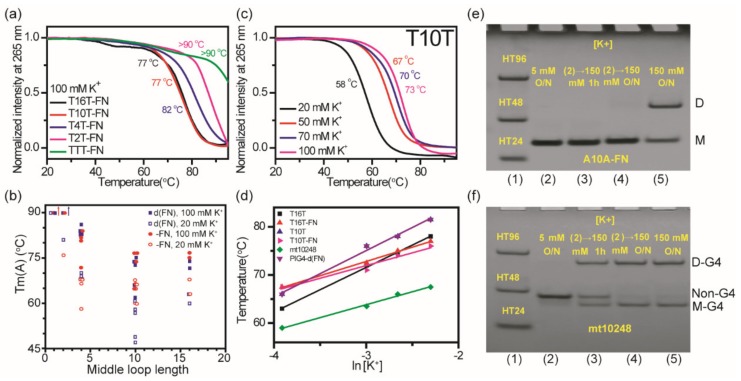
The effect of K^+^ concentration on thermal stability of (G_3_HG_3_N_m_G_3_HG_3_) G4 structures. (**a**) CD melting curves of T16T-FN, T10T-FN, T4T-FN T2T-FN, and TTT-FN after the addition of 100 mM K^+^ overnight. (**b**) The plots of Tm(A) as a function of middle loop length of both (G_3_TG_3_N_m_G_3_TG_3_) and (G_3_TG_3_N_m_G_3_TG_3_)-FN sequences after the addition of 20 mM K^+^ and 100 mM K^+^ overnight. (**c**) CD melting curves of T10T after the addition of 20, 50, 70, and 100 mM K^+^ overnight. (**d**) The plots of Tm(A) of T16T, T16T-FN, T10T, T10T-FN, PIG4-d(FN), and mt10248 measured from CD melting as a function of ln[K^+^] with linear regression fits. (**e**) PAGE assays of marker bands of HT24 (T_2_AG_3_)_4_, HT48 (T_2_AG_3_)_8_, and HT96 (T_2_AG_3_)_16_ (lane 1) and A10A-FN after the addition of 5 mM K^+^ overnight (lane 2), followed by the addition of K^+^ to 150 mM for 1 h (lane 3) and overnight (lane 4), and A10A-FN after the addition of 150 mM K^+^ overnight (lane 5). (**f**) PAGE assays of marker bands of HT24 (T_2_AG_3_)_4_, HT48 (T_2_AG_3_)_8_, and HT96 (T_2_AG_3_)_16_ (lane 1) and mt10248 after the addition of 5 mM K^+^ overnight (lane 2), followed by the addition of K^+^ to 100 mM for 1 h (lane 3) and overnight (lane 4), and mt10248 after the addition of 100 mM K^+^ overnight (lane 5). The same DNA concentration of 100 μM was used in the experiments of CD and PAGE of this work.

We further measured melting temperature for G4 unfolding of these sequences after the addition of K^+^ overnight without annealed to examine the effect of K^+^ concentration on monomeric and dimeric G4 structures. Figure 8a shows CD melting curves at 265 nm for T16T-FN, T10T-FN, T4T-FN, T2T-FN, and TTT-FN in 100 mM K^+^ solution. Since the thermal melting curve may not be at thermodynamic equilibrium, such melting temperatures are named apparent melting temperatures [31] or transition temperatures [32]. The apparent melting temperature, Tm(A), was obtained from the first derivative of the melting curve and was listed in Table 1. Figure 8b shows plots of Tm(A) as a function of middle loop length of both (G_3_HG_3_N_m_G_3_HG_3_) and (G_3_HG_3_N_m_G_3_HG_3_)-FN sequences after the addition of 20 mM K^+^ and 100 mM K^+^ overnight. Consistent with the previous finding [7,8,9], Tm(A) generally decreases as the middle loop length increases up to 10 bases. In addition, the single base loop with thymine has higher Tm(A) than the single base loop with adenine.

The Tm normally increases as the K^+^ concentration increases, indicating that the G4 structure is more stable at high K^+^ concentration. Previously, a linear plot of Tm vs ln[K^+^] with a slope about ~7.0 was obtained for a number of G4 structures [6,33]. Here we measured Tm(A) of T16T, T10T, T16T-FN, and T10T-FN together with native G-rich sequences of PIG4-d(FN) and mt10248 as a function of K^+^ concentration to compare the effect of K^+^ concentration on monomeric and dimeric G4 structures. Figure 8c shows the CD melting curves of T10T in 20 mM, 50 mM, 70 mM, and 100 mM K^+^ solution. Figure 8d shows the plots of Tm(A) of these six sequences as a function of ln [K^+^] with the linear regression fits. Here the slope near 9.0 was obtained for dimeric G4 structures of T16T, T10T, and PIG4-d(FN), while the slope near 6.0 was obtained for monomeric G4 structures of T16T-FN, T10T-FN, and mt10248. It is suggested that high K^+^ concentration generally plays a more crucial role in dimeric G4 formation. 

Previously, we have studied the effect of K^+^ concentration on structural conversion of intramolecular and intermolecular G4s of bcl2mid [34]. The intramolecular G4 formation of bcl2mid is predominant in 5 mM K^+^ solution and the intermolecular G4 formation appears in 20 mM K^+^ solution. However, the mix of bcl2mid in 5 mM K^+^ solution with an equal amount of the 150 mM K^+^ solution showed no discernible change from intramolecular G4 structure to intermolecular G4 structure at room temperature, suggesting that the dimeric G4 formation is not due to the aggregation of two monomeric G4 structures. Here the imino proton NMR signals of A10A-FN appreared almost identical in the 10.0−12.5 ppm region in 20 mM K^+^ solution and in 100 mM K^+^ solution. Notably, the PAGE results showed a marked increase in dimeric G4 population of A10A-FN in 100 mM K^+^ than in 20 mM K^+^ solution. Is it possible that high K^+^ concentration promotes the stacking of two monomeric G4 structures? The Tm(A) of A10A-FN measured from the CD melting curve at 265 nm is 56 °C in 5 mM K^+^ solution (data not shown). The PAGE results showed no dimeric formation of A10A-FN in 5 mM K^+^ solution and no discernible change from monomer to dimer after the addition of K^+^ to 150 mM K^+^ solution overnight (Figure 8e), implying that the dimeric formation is not simply promoted by the stacking of two monomeric G4 structures in 100 mM K^+^ solution. 

In contrast, the PAGE results of mt10248 showed very small amounts of monomeric G4 formation and large amounts of non-G4 residues in 5 mM K^+^ solution overnight. The significant decrease in non-G4 residues together with the marked increase of monomeric and dimeric G4 populations are detected after the addition of K^+^ to 100 mM K^+^ solution (Figure 8f). Consistent with PAGE results, the imino proton NMR signals of mt10248 in 5 mM K^+^ solution are identical as in 20 mM K^+^ solution, and the imino proton NMR signals of mt10248 after further addition of K^+^ to 100 mM K^+^ solution are the same as in 100 mM K^+^ solution (Figure S6). Since a G-tract is involved in WC hydrogen bond formation characterized by the imino proton signals near 13 ppm, it appears that mt10248 forms interlocked G4 dimer in 100 mM K^+^ solution [35].

### 2.8. Ligand Binding to (G_3_TG_3_N_m_G_3_TG_3_)-FN G4 Structures

**Figure 9:**
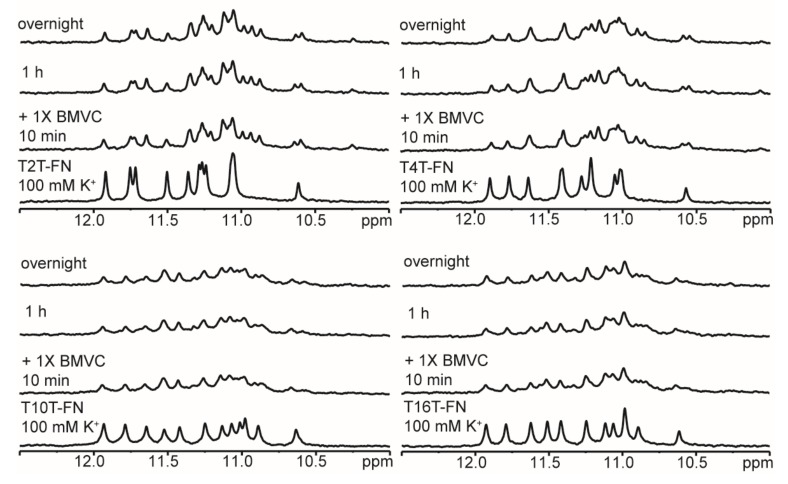
Ligand binding of BMVC to (G_3_TG_3_N_m_G_3_TG_3_)-FN G4 structures. Imino proton NMR spectra of T2T-FN, T4T-FN, T10T-FN, and T16T-FN in 100 mM K^+^ solution and after 10 min, 1 h, and overnight addition of 1 eq. BMVC.

Very recently, Liu et al. [26] reported ligand binding of BMVC to match the G-quartet for an optimal stacking interaction with Pu22 (i.e., T2T-FN). Given that the NMR spectra of T2T-FN and T4T-FN are very similar, it is of interest to examine whether the spectral patterns of T2T-FN and T4T-FN upon interaction with BMVC are also similar. In addition, the imino proton NMR signal in the region of 10.0−12.5 ppm of T10T-FN and T16T-FN are almost identical. Figure 9 shows the imino proton NMR spectra of T2T-FN, T4T-FN, T10T-FN, and T16T-FN upon interaction with 1 equivalent of BMVC in 100 mM K^+^ solution. The results suggested that these (G_3_TG_3_N_m_G_3_TG_3_)-FN G4 structures have similar binding sites for BMVC, supporting some structural similarity involved in these sequences. Similar NMR results were also detected in T2T-FN and T4T-FN upon interaction with 1 equivalent of BRACO-19 in 100 mM K^+^ solution (Figure S7). Such similarity is crucial for drug development to target G4 structures. 

## 3. Discussion

A G-rich sequence containing four G-tracts with ≥2 guanines and three loops to connect G-tracts can possibly form a G4 structure. Although there are different types of G4 structures, they all involve the stacking of G-quartets in the formation of G4 structures. Thus, loop sequence plays a major role on their folding topology and thermal stability [4,5]. To deduce rules for predicting the folding pattern and stability of G4s, the effects of loop length and base composition on G4 stability have been extensively studied [6,7,8,9,10,11,12]. Most of studies used CD spectra to identify different types of G4 structures and melting to measure melting temperature for characterizing G4 stability [6,7]. For example, CD spectra suggested G-rich sequences (G_3_N_i_G_3_N_j_G_3_N_k_G_3_) with a total loop length of not more than five nucleotides (i + j + k ≤ 5) favor parallel G4 structures, while G-rich sequences with a total loop length of more than five nucleotides could form nonparallel G4 structures in 20 mM K^+^ solution [7]. The Tm(A) of (G_3_HG_3_N_m_G_3_HG_3_) generally decreases when the length of the middle loop is increased. In addition, the loop base with a single T-base has higher Tm than a single A-base [6,7,8]. Smargiasso et al. [8] used gel electrophoresis and mass analysis to demonstrate that sequences with short loops, such as H1H, H2H, and H4H, form predominantly dimers and trimers in 150 mM K^+^ solution. Particularly, sequences having a single base in their third loop systematically form high-order oligomers. They further proposed that the formation of dimers and trimers are likely due to the stack of parallel intramolecular G4 structures, although some contribution of interlocked dimers cannot be ruled out. Moreover, they reported that monomers are favored when flanking nucleotides are added [8]. Generally, such studies on G4 structures were conducted at low strand concentrations, for example 5 μM, which were not sufficient to provide NMR spectra for the comparison of their G4 structures. 

### 3.1. Structural Diversity and Similarity of (G_3_HG_3_N_m_G_3_HG_3_) Sequences with and without Flanking Nucleotides

In this work, we have first used CD, NMR, and PAGE to study G4 formation of TTT, T2T, T4T, T10T, and T16T without flanking nucleotides after the addition of K^+^. The imino proton NMR spectra of TTT and T2T are similar, but different from T4T and very different from T10T and T16T. The lack of distinct imino proton signals in these NMR spectra is a great challenge for structural analysis. The PAGE results suggest that sequences with long length in the middle loop favor to form dimeric G4 structures, while sequences with short length in the middle loop tend to form high-order G4 structures. Of interest is that PAGE assays show two close bands in TTT, but one band in T2T. Previously, the mass analysis of 5 μM H1H illustrated the presence of dimeric and trimeric G4 structures in 150 mM K^+^ solution [8]. Given that the 100 μM TTT may form even high-order G4 structure, we propose two interlocked G4 topologies (Figure 1a). This is because the two close bands in gel migration are unlikely due to the stack of parallel monomeric G4 structures. At present, we are not able to rule out the possible contribution from stack G4 topology (Figure 1b). In addition, the one band, not two bands, in T2T is probably due to the mismatch of TA middle loop to other T loops.

Here most of the CD spectra of (G_3_TG_3_N_m_G_3_TG_3_) sequences with or without flanking nucleotides show similar positive band at 265 nm as a character of parallel G4 structure after the addition of K^+^. The PAGE results clearly indicate that those sequences without flanking nucleotides favor to form multimeric G4 structures, while those sequences with flanking nucleotides tend to form monomeric G4 structures. Indeed, Rachwal et al. [33] previously found a monomeric parallel G4 formation of (TG_3_TG_3_TG_3_TG_3_T) in K^+^ solution (Figure 1c). We consider that this monomeric G4 structure is also the G4 structure formed by TTT-FN in this work. In addition, Ambrus et al. [25] have determined the monomeric G4 structure of Pu22 (T2T-FN) in K^+^ solution (Figure 1c). This finding is important since monomeric G4 structure is more biologically relevant. In addition, their imino proton NMR spectra allow us to examine the effects of loop composition and flanking nucleotides on their G4 structures. Different NMR spectra due to the change of base composition in the middle loop indicate that loop composition plays an important role in determining their G4 structures. Surprisingly, we found that the imino proton NMR spectral patterns of T10T-FN, A10A-FN, C10C-FN are almost identical, and the imino proton NMR spectral patterns of T4T-FN, A4A-FN, and C4C-FN are also very similar in 20 mM K^+^ solution, implying that the change of a single base T, A, and C in loop 1 and loop 3 in (G_3_HG_3_N_m_G_3_HG_3_)-FN sequences with the same middle loop has no appreciable effect on their monomeric G4 structures. 

### 3.2. Effects of Intramolecular and Intermolecular WC Hydrogen Bonds on G4 Structures

Recently, Lim and Phan [30] used NMR to investigate a number of quadruplex-duplex mixed structures. Particularly, the imino proton NMR spectrum of a construct V sequence, 5′-TTG_3_TG_3_CGC GAAGCATTCGCGG_3_TG_3_T-3′, showed two peaks near 13.5 ppm for T(19) and T(20), three peaks near 13 ppm for G(11), G(13), and G(22), and twelve peaks for G4 signals, such imino proton NMR signals were valuable to propose a hybrid quadruplex-duplex structure with a hairpin duplex formed within the long middle loop of monomeric G4 structure (Figure 1d) [30]. Onel et al. reported two similar quadruplex-duplex mixed structures (Figure 1d,e) coexisted in a native PIG4 sequence located at the upstream of the human BCL2 gene P1 promoter [29]. In addition, Krauss et al. [36] have introduced an 8-mer, d-(TAACGCTA), complementary to the 3′ end of a long sequence, 5′-G_3_TG_3_TG_3_TG_3_TTAGCGTTA-3′, for revealing in detail the quadruplex-duplex interface using X-ray crystallography. They found that such interface could provide a stable pocket for the binding of selective molecules. Better understanding of such quadruplex-duplex junction may be important in biological and therapeutic implications [15] and in DNA nanotechnology [37]. 

It is noted that the middle loop in T16T and T16T-FN may form either intramolecular or intermolecular hydrogen bonds in 10 mM Tris. After the addition of K^+^, T16T forms dimeric G4 structure and T16T-FN adopts into monomeric G4 structure. Considering that both T16T-FN and construct V sequence [30] contain a duplex hairpin in the middle loop, it is very likely that T16T-FN can also form a monomeric quadruplex-duplex structure (Figure 1d). Since the imino proton signals in the region of 12.5−14.0 ppm of T16T and T16T-FN are almost identical, such signals are due to intramolecular WC hydrogen bond. Thus, two possible topologies of dimeric quadruplex-duplex junction are proposed for T16T (Figure 1f). Here our results indicate that intramolecular hydrogen bond is a prior formation than intermolecular hydrogen bond in T16T-FN.

Although the middle loop in T10T may form intermolecular WC hydrogen bonds, the imino proton NMR signals of T10T due to WC hydrogen bonds are not detected after the addition of K^+^. Here two possible topologies of dimeric G4 structures are proposed for T10T (Figure 1g). Of interest is that the imino proton NMR signals in the region of 12.5−14.0 ppm of T10T-FN are clearly detected in 100 mM K^+^ solution, but not detected in 20 mM K^+^ solution. Notably, the imino proton NMR signals in the region of 10.0−12.5 ppm are almost identical in 20 mM K^+^ and in 100 mM K^+^ solution. Such similar spectral features are also observed in A10A-FN and C10C-FN. The imino proton NMR signals due to WC hydrogen bonds in the region of 12.5−14 ppm of T10T-FN and T16T-FN are different. According to the study of quadruplex-duplex junction by Lim and Phan [30], we can assign the imino proton NMR signal detected near 13.5 ppm to T(13) in T10T-FN. Such signal cannot be due to intramolecular WC hydrogen bond, instead intermolecular hydrogen bond of T10T-FN. Thus, the weak imino proton signals near 13.5 ppm corresponding to a weak band in PAGE is likely due to a dimeric duplex of T10T-FN. In addition, we propose that T10T-FN forms a monomeric G4 structure (Figure 1c). 

Considering the formation of intramolecular hydrogen bonds in T16T-FN and intermolecular hydrogen bonds in T10T-FN, it is surprised that the imino proton signals of T10T-FN and T16T-FN in the region of 10.0−12.5 ppm are almost identical for monomeric G4 structures. For comparison, we further study the NMR spectra of T10T-1-FN and A10A-1-FN, a single base substitution of A-base by T-base in the middle loop of H10H-FN. Indeed, the NMR results show no imino proton signal in the region of 12.5−14 ppm, but almost identical imino proton signals in the region of 10.0−12.5 ppm to that of T10T-FN and A10A-FN in 100 mM K^+^ solution (Figure S8). They also form a monomeric G4 structure (Figure 1c). Here our results illustrate that such different WC hydrogen bonds play no appreciable change in their monomeric G4 structures.

### 3.3. Effects of Potassium Concentration on (G_3_HG_3_N_m_G_3_HG_3_)-FN G4 Structures

The importance of K^+^ concentration to the G-rich sequences is not only to stabilize the G4 structure but also to cause changes in structural populations and polymorphism. Previously, we found that mt10251 sequence without flanking nucleotides can form intramolecular WC hydrogen bonds in 10 mM Tris. After the addition of 20 mM K^+^, the intermolecular G4 formation was much more dominated than the intramolecular G4 formation [24]. Here NMR results show that the intramolecular WC hydrogen bonds in a native mt10248 sequence is negligible in 10 mM Tris. After the addition of 20 mM K^+^, NMR results show that the hairpin formation is prior to the G4 formation. Notably, PAGE results show that only a small portion of mt10248 form monomeric G4 structures in 20 mM K^+^ solution, however, more dimeric G4 population than monomeric G4 population is detected in 100 mM K^+^ solution. Of importance is that the arising time of G4 formation characterized by the 265 nm CD signal is much shorter after the addition of 100 mM K^+^ than the addition of 20 mM K^+^, implying that high K^+^ concentrations kinetically favor to dimeric G4 formation. In addition, PAGE results show that a large portion of non-G4 residues are detected in 20 mM K^+^ solution but are almost negligible in 100 mM K^+^ solution. The study of numerous single-base mutants of 10251 indicated that the third G-tract is involved in hairpin formation with three consecutive C-bases [24,35]. Considering similar population of monomeric G4 formation of 10248 in 20 mM K^+^ and in 100 mM K^+^ solution, such hairpin formation must be unfolded for monomeric G4 formation (Figure 1c).

The PAGE assays of A10A-FN prepared in 5 mM K^+^ solution overnight and then adding more K^+^ to 150 mM K^+^ solution show no dimeric formation, which is very different from the preparation of A10A-FN in 100 mM K^+^ solution. In contrast, the PAGE assays of mt10248 prepared in 5 mM K^+^ solution overnight and then adding more K^+^ to 100 mM K^+^ solution show both monomeric and dimeric formation, which is almost the same as the preparation of mt10248 in 100 mM K^+^ solution. This is because only a small portion of mt10248 form monomeric G4 structure in 5 mM K^+^ solution and a large portion of residues favor to form dimeric G4 structure after the addition of K^+^ to 100 mM K^+^. However, most of A10A-FN form monomeric G4 structure in 5 mM K^+^ solution. As a result, there is no dimeric formation after the addition of K^+^ to 150 mM K^+^ solution. Apparently, significant increase in dimeric population of A10A-FN in 100 mM K^+^ solution is not due to aggregation of monomeric G4 structure.

Considering the earlier detection of hairpin structure in the NMR spectra of mt10248 after the addition of 20 mM K^+^, the formation of WC hydrogen bond is kinetically prior to the formation of Hoogsteen hydrogen bond. However, the slowly decrease of imino proton signals near 13.5 ppm together with the gradual increase of imino proton signals in the region of 10−12.5 ppm as a function of time suggests that the hairpin unfolding is faster than G4 unfolding, implying that monomeric G4 structure is more thermal stable than the hairpin structure [38,39]. Indeed, the Tm(A) for hairpin structure is 54.4 ± 0.5 °C for hairpin structure of a mt10251 mutant [35] and 58 ± 1.06 °C for monomeric G4 structure of 10248 in 20 mM K^+^ solution. We further found more population of dimeric G4 formation than monomeric G4 formation of mt10248 in 100 mM K^+^ solution, implying that high K^+^ concentration favors the formation of dimeric than monomeric G4 structures. Previously, we found that the possible formation of dimeric quadruplex-duplex structure of mt10251 (Figure 1h) can be ignored [35]. Thus, two possible topologies of dimeric G4 structures without hairpin formation are proposed for T10T (Figure 1g). Consistent with PAGE results, the imino proton signals near 13.5 ppm is likely due to a monomeric hairpin structure, the fine signals and the broad signals overlapped in the region of 10−12.5 ppm are due to monomeric and dimeric G4 structures, respectively.

### 3.4. Implication to Native Sequences

The findings that the (G_3_HG_3_N_m_G_3_HG_3_) sequences without four consecutive G-bases favor to form multimeric G4 structures and the (G_3_HG_3_N_m_G_3_HG_3_)-FN sequences tend to form monomeric G4 structures can be applied to native G-rich sequences, such as mt10248 [24], Pu22 [25], GTERT [28], and PIG4 [29]. In addition, conformational change from a monomeric G4 formation to a multimeric G4 formation is detected in T4T and T4T-3 after the addition of 100 mM K^+^. Such conformational change is also observed in GTERT-d(FN) after the addition of 100 mM K^+^. Notably, they all show spectral change from a major 265 nm band together with a minor 290 nm band to a single 265 nm band after the addition of 100 mM K^+^ from 1 h to overnight. The detection of 290 nm CD band is normally due to nonparallel G4 formation. The absence of the 290 nm band in the final state suggests that this nonparallel G4 structure is an intermediate state. The PAGE results indicate that they all involve conformational change from monomeric G4 to multimeric G4 structures. It is important to determine their initial and final G4 structures. In addition, the similarity of such conformational change from monomeric to multimeric G4 structures deserve further study. The study of underlying mechanism of conformational change is not only important to explore the complexity and beauty of DNA but also useful to design DNA sequences for nanomaterial application.

The monomeric G4 structure formed by *chl1*-d(FN) can be described by four consecutive G bases in the second and the third G-tracts, which can change a single base to two bases in loop 1 and loop 3. Of interest is that the human *chl1* sequence forms an unprecedented G4 structure in K^+^ solution [27]. The twelve imino proton NMR signals of *chl1* detected in the 11−12 ppm region suggest that it is composed of three G-quartets. Spectral analysis revealed that the first G base is positioned within the central G-quartet. In addition, a V-shaped loop, spanning three G-quartet planes, containing no bridging nucleotides. As a result, the *chl1* sequence adopts an unusual G_2_-G_3_-G_4_-G_2_ alignment of G-tracts with four loops of 2, 3, 0, and 1 loop lengths. In this work, we also found similar twelve imino proton NMR signals of *chl1*-d(FN) in the region of 11−12 ppm. However, the imino proton signal of G(11) at 11.0 ppm detected in *chl1* is not observed in *chl1*-d(FN). Given that the only difference between *chl1* and *chl1*-d(FN) is the deletion of T(19) in *chl1*-d(FN), the G4 structure of *chl1*-d(FN) deserves further study for the comparison with the G4 structure of *chl1*.

### 3.5. Similarity for the Development of G4 Binding Ligands

One of the purposes of studying the structural diversity and similarity of G4 structures is to search for common features for the development of G4 binding ligands. Here similar NMR spectral patterns of ligand binding of BMVC to T2T-FN (Pu22), T4T-FN, T10T-FN and T16T-FN suggest the similarity of these G4 structures, implying that the (G_3_HG_3_N_m_G_3_HG_3_)-FN sequences provide similar binding sites for G4 ligands. Further study of ligand binding of BMVC to *chl1* and *chl1*-d(FN) may validate the implication of this work. In addition, similar NMR spectral patterns of ligand binding of BRACO-19 as BMVC to T2T-FN (Pu22) and T4T-FN suggest that these sequences afford the same binding sites for BRACO-19 and BMVC. Previously, we used time-gated fluorescence lifetime imaging microscopy of a BMVC derivative to visualize G4 foci in cells [23]. In addition, we found the decrease in the number of G4 foci in the pretreatment of HeLa cells with BRACO-19, suggesting that BRACO-19 directly binds to G4s in cells [40]. Thus, the study of ligand binding in this work further support our previous work on G4s in cells. 

## 4. Materials and Methods 

### 4.1. DNA Preparation

DNA oligonucleotides were purchased from Bio Basic (Markham, ON, Canada) and dissolved in 10 mM Tris (pH 7.5). They were then subjected to heat-denaturation at 95 °C for 10 min and annealed to room temperature at a rate of 1 °C/min. The annealed oligonucleotides were stored at 4 °C overnight until the further experiments. The DNA concentrations were determined using a UV-Vis absorption spectrometer (Implen, Munich, Germany).

### 4.2. Circular Dichroism (CD)

CD experiments were conducted using a spectropolarimeter (J-815, Jasco, Tokyo, Japan) with a bandwidth of 2 nm, at a scan speed of 50 nm/min and a step resolution of 0.2 nm over a spectral range of 210–350 nm. The DNA concentration in each sample was 100 μM dissolved in 10 mM Tris (pH 7.5), and a stock solution of 3 M KCl (Sigma-Aldrich, St. Louis, MO, USA) was added to the DNA samples to attain a final K^+^ concentration. The observed signals were baseline subtracted. The melting curves were recorded at 265 nm from 20 to 95 °C with a temperature ramping rate of 1 °C/min rate controlled by a Peltier thermal coupler chamber (PFD-425S/15, Jasco).

### 4.3. Nuclear Magnetic Resonance (NMR) Spectroscopy

NMR experiments were performed at 25 °C on an AVIII 500 MHz spectrometer (Bruker, Rheinstetten, Germany) equipped with a Prodigy probehead. One-dimensional (1D) imino proton NMR spectra were recorded using a WATERGATE for water suppression. The strand concentrations of the NMR samples were typically 100 μM, containing 10% D_2_O in 10 mM Tris (pH 7.5) or specific K^+^ conditions, with an internal reference of 0.01 mM 4,4-dimethyl-4-silapentane-1-sulfonic acid (DSS).

### 4.4. Polyacrylamide Gel Electrophoresis (PAGE)

PAGE was conducted using 20 % polyacrylamide and 0.5× TBE gels. The DNA concentration for each sample was 100 μM, that is, the same concentration used for CD and NMR. PAGE was conducted at 150 V for 3.5 h at 4 °C. The gels were then photographed under ultraviolet (UV) light at 254 nm using a digital camera.

## 5. Conclusions

In this work, we used NMR, CD, and PAGE to study the effects of loop length and base composition on G-rich (G_3_HG_3_N_m_G_3_HG_3_) sequences with and without flanking nucleotides. Since such (G_3_HG_3_N_m_G_3_HG_3_) configuration could be found in a number of native DNA sequences, we attempted to establish the correlation between model sequences and native sequences to deduce the structural similarity from these sequences. For example, we found that (G_3_HG_3_N_m_G_3_HG_3_) sequences tend to form multimeric G4 structures, while (G_3_HG_3_N_m_G_3_HG_3_)-FN sequences favor to form monomeric G4 structures. Such findings can be found in native sequences of mt10248, PU22, GTERT, and PIG4. However, some important findings in model sequences have not been tested in native sequences, such as a single-base difference of A, T, and C in loop 1 and loop 3 in (G_3_HG_3_N_m_G_3_HG_3_) sequences showed no obvious change on their G4 structures in this work. We further examined such similarity by using G4 binding ligands, which may be useful for the use and design of G4 ligands in human cancer. Considering a large amounts of G-rich sequences in human gene, this work demonstrates a possible approach to find structural similarity from G-rich sequences.

## Figures and Tables

**Table 1:** Sequences studied in this work and their melting temperatures and conformations in the presence of 20 and 100 mM K^+^, respectively.

	Sequence(5′→3′)	Tm(A) (°C, 20 mM K^+^)	Tm(A) (°C, 100 mM K^+^)
T16T	GGGTGGGTTGCGCAATTGCGCTTGGGTGGG	63 ± 1.55 (D, wM) ^a^	78 ± 0.65 (D, wM)
T10T	GGGTGGGTTGCATGCTTGGGTGGG	58 ± 1.44 (D)	73 ± 1.12 (D)
T4T	GGGTGGGCATGGGGTGGG	68 ± 0.30 (M→H) ^b^	84 ± 1.56 (M→H)
T2T	GGGTGGGTAGGGTGGG	81 ± 0.99 (H)	>90 (H)
TTT	GGGTGGGTGGGTGGG	62 ± 1.70, >90	78 ± 0.92, >90
T4T-1	GGGTGGGATTAGGGTGGG	69 ± 0.92 (H)	85 ± 0.77 (H)
T4T-2	GGGTGGGCATCGGGTGGG	68 ± 1.61 (H)	83 ± 0.62 (H)
T4T-3	GGGTGGGACGTGGGTGGG	70 ± 0.49 (M→H)	86 ± 1.51 (M→H)
T16T-FN	TGAGGGTGGGTTGCGCAATTGCGCTTGGGTGGGTAA	68 ± 0.25 (M)	77 ± 0.49 (M)
T10T-FN	TGAGGGTGGGTTGCATGCTTGGGTGGGTAA	68 ± 1.02 (M, wD)	77 ± 0.62 (M, wD)
T4T-FN	TGAGGGTGGGCATGGGGTGGGTAA	68 ± 0.44 (M)	82 ± 0.35 (M, wD)
T2T-FN	TGAGGGTGGGTAGGGTGGGTAA	76 ± 0.55 (M)	>90 (M)
TTT-FN	TGAGGGTGGGTGGGTGGGTAA	>90 (M)	>90 (M)
A16T	GGGAGGGTTGCGCAATTGCGCTTGGGTGGG	- (D)	74 ± 1.21 (D)
A10A	GGGAGGGTTGCATGCTTGGGAGGG	47 ± 1.10 (D)	62 ± 0.38(D)
C10C	GGGCGGGTTGCATGCTTGGGCGGG	57 ± 1.20 (D)	74 ± 1.10 (D)
T10T-1	GGGTGGGTTGCTTGCTTGGGTGGG	61 ± 0.60 (D)	76 ± 0.46 (D)
A10A-1	GGGAGGGTTGCTTGCTTGGGAGGG	49 ± 0.86 (D)	66 ± 1.12 (D)
A16T-FN	TGAGGGAGGGTTGCGCAATTGCGCTTGGGTGGGTAA	- (M, wD)	75 ± 0.29 (M, wD)
A10A-FN	TGAGGGAGGGTTGCATGCTTGGGAGGGTAA	60 ± 0.13 (M, wD)	66 ± 0.42 (M, D)
C10C-FN	TGAGGGCGGGTTGCATGCTTGGGCGGGTAA	65 ± 0.42 (M, wD)	74 ± 1.16 (M, D)
T10T-1-FN	TGAGGGTGGGTTGCTTGCTTGGGTGGGTAA	67 ± 0.28 (M)	77 ± 0.31 (M)
A10A-1-FN	TGAGGGAGGGTTGCTTGCTTGGGAGGGTAA	59 ± 0.13 (M)	68 ± 0.64 (M)
A4A-FN	TGAGGGAGGGCATGGGGAGGGTAA	58 ± 0.36 (M, wD)	73 ± 0.62 (M, D)
C4C-FN	TGAGGGCGGGCATGGGGCGGGTAA	68 ± 0.48 (M)	81 ± 0.74 (M, wD)
T4T-1-FN	TGAGGGTGGGATTAGGGTGGGTAA	67 ± 1.63 (M)	83 ± 0.95 (M, wD)
mt10248	CCAGGGTGGGAGTAGTTCCCTGCTAAGGGAGGGTAG	58 ± 1.06 (M, wD)	67 ± 1.08 (D, wM)
*chl1*-d(FN)	GGGTGGGGAAGGGGTGGG	71 ± 0.60	63 ± 0.39, 84 ± 0.61 (M)
GTERT-d(FN)	GGGAGGGGCTGGGAGGG	70 ± 0.66	84 ± 1.48 (M→H)
PIG4-d(FN)	GGGCGGGAGCGCGGCGGGCGGGCGGG	-	82 ± 1.52 (D)

^a^ M: monomer, D: dimer, H: high-order, w: weak. ^b^ M→D: monomer converts to dimer as a function of time.

## Supplementary Materials

The following are available online, Figure S1: Kinetic study of G4 formation. The kinetic traces of T10T, T4T-1, and T2T for multimeric G4 formations and T10T-FN, T4T-1-FN, and T2T-FN for monomeric G4 formations by monitoring the 265 nm CD signal after the addition of 20 mM K^+^. Figure S2: The effect of loop base on G4 formation of (G_3_HG_3_N_m_G_3_HG_3_) sequences. CD and NMR spectra of A16T, A10A, and C10C in 10 mM Tris and after 1h and overnight addition of 100 mM K^+^ together with their PAGE assays. Figure S3: The effect of loop base on G4 formation of (G_3_HG_3_N_m_G_3_HG_3_) sequences with flanking nucleotides. NMR spectra of A16T-FN, A10A-FN and C10C-FN in 10 mM Tris and after 1h and overnight addition of 100 mM K^+^ together with their PAGE assays. The same DNA concentration of 100 μM was used in the experiments of NMR and PAGE of this work. Figure S4: The effect of loop base on G4 formation of (G_3_HG_3_N_4_G_3_HG_3_) sequences with flanking nucleotides. NMR spectra of A4A-FN and C4C-FN in 10 mM Tris and after 1h and overnight addition of 100 mM K^+^ together with their PAGE assays. The same DNA concentration of 100 μM was used in the experiments of NMR and PAGE of this work Figure S5: The effect of loop base on G4 formation of (G_3_T_2_G_3_N_4_G_3_TG_3_) and (G_3_T_2_G_3_N_4_GT_2_G_3_) sequences. CD and NMR spectra of T_2_4T and T_2_4T_2_ in 10 mM Tris and after 1h and overnight addition of 100 mM K^+^ together with their PAGE assays after overnight addition of 100 mM K^+^. The same DNA concentration of 100 μM was used in the experiments of CD, NMR and PAGE of this work. Figure S6: The effect of K^+^ concentration on mt10248 G4 structures. The imino proton NMR spectra of mt10248 in 5 mM K^+^ solution overnight and after 10 min, 1 h, and overnight addition of K^+^ to 100 mM K^+^ solution. Figure S7: Ligand binding of BRACO-19 to (G_3_TG_3_N_m_G_3_TG_3_)-FN G4 structures. Imino proton NMR spectra of T2T-FN and T4T-FN in 100 mM K^+^ solution and after 10 min, 1 h, and overnight addition of 1 eq. BRACO-19. Figure S8: The effect of loop base on G4 formation of (G_3_HG_3_N_10_G_3_HG_3_)-FN sequences. NMR spectra of A10A-1-FN and T10T-1-FN in 10 mM Tris and after 1h and overnight addition of 20 mM K^+^ and 100 mM K^+^.
