# Supplementary material for: Effects of Length and Loop Composition on Structural Diversity and Similarity of (G3TG3NmG3TG3) G-Quadruplexes
Source: Molecules. 2020 Apr 13;25(8):1779. doi: 10.3390/molecules25081779 (PMC7221631; doi:10.3390/molecules25081779)
Supplement: Supplementary file 1 [file molecules-25-01779-s001.pdf]

molecules

Supplementary Materials: Effects of Length and Loop Composition on Structural Diversity and Similarity of $(G_3TG_3N_mG_3TG_3)$ G-Quadruplexes

Jie Li,[+] I-Te Chu,[+] Ting-An Yeh, De-Yu Chen, Chiung-Lin Wang and Ta-Chau Chang,[*]

Institute of Atomic and Molecular Sciences, Academia Sinica, Taipei 106, Taiwan
[+] Contributed equally to this work
[*] Correspondence: tcchang@po.iams.sinica.edu.tw

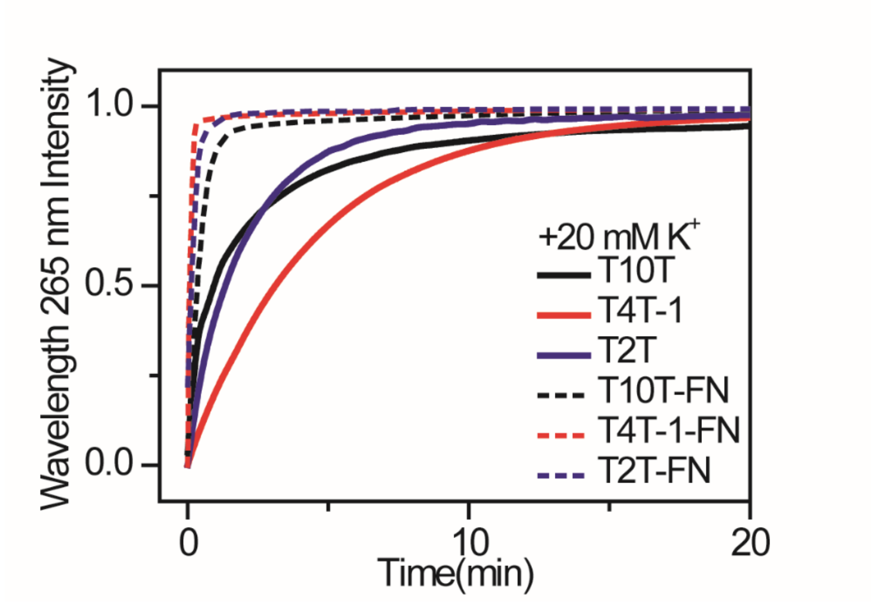

Figure S1. Kinetic study of G4 formation. The kinetic traces of T10T, T4T-1, and T2T for multimeric G4 formations and T10T-FN, T4T-1-FN, and T2T-FN for monomeric G4 formations by monitoring the 265 nm CD signal after the addition of 20 mM K^+.

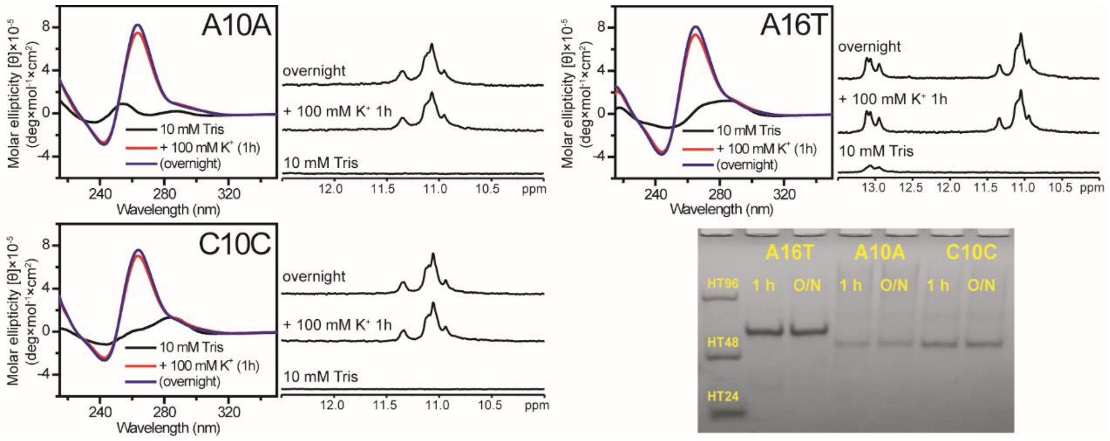

Figure S2. The effect of loop base on G4 formation of $(G_3HG_3N_mG_3HG_3)$ sequences. CD and NMR spectra of A16T, A10A, and C10C in 10 mM Tris and after 1h and overnight addition of 100 mM K^+ together with their PAGE assays.

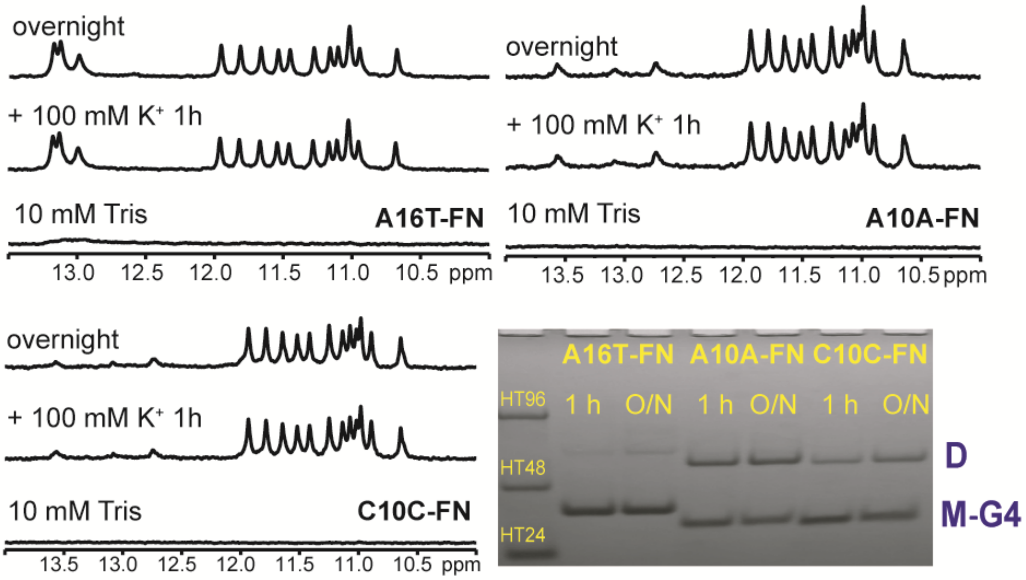

Figure S3. The effect of loop base on G4 formation of ($G_3HG_3N_mG_3HG_3$) sequences with flanking nucleotides. NMR spectra of A16T-FN, A10A-FN and C10C-FN in 10 mM Tris and after 1h and overnight addition of 100 mM K^+ together with their PAGE assays. The same DNA concentration of 100 μM was used in the experiments of NMR and PAGE of this work.

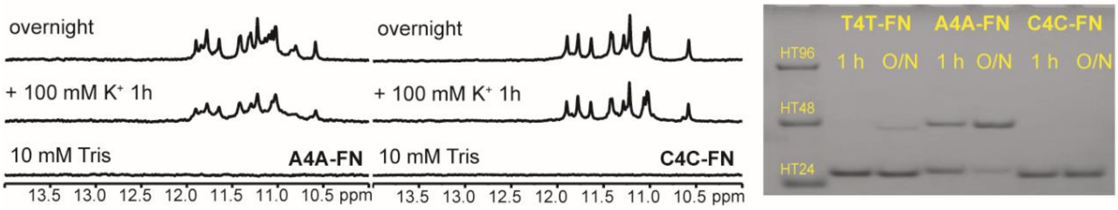

Figure S4. The effect of loop base on G4 formation of ($G_3TG_3N_4G_3TG_3$) sequences with flanking nucleotides. NMR spectra of A4A-FN and C4C-FN in 10 mM Tris and after 1h and overnight addition of 100 mM K^+ together with their PAGE assays. The same DNA concentration of 100 μM was used in the experiments of NMR and PAGE of this work.

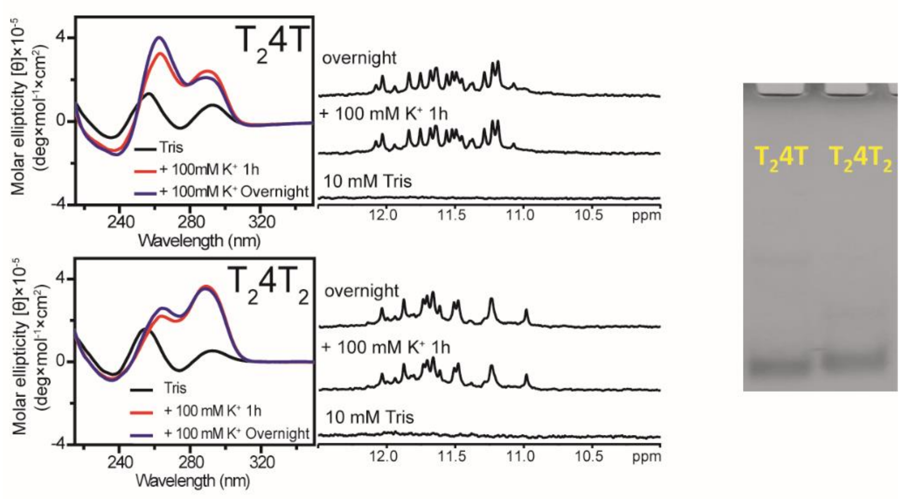

Figure S5. The effect of loop base on G4 formation of ($G_3T_2G_3N_4G_3TG_3$) and ($G_3T_2G_3N_4GT_2G_3$) sequences. CD and NMR spectra of T_24T and T_24T_2 in 10 mM Tris and after 1h and overnight addition of 100 mM K^+ together with their PAGE assays after overnight addition of 100 mM K^+. The same DNA concentration of 100 μM was used in the experiments of CD, NMR and PAGE of this work.

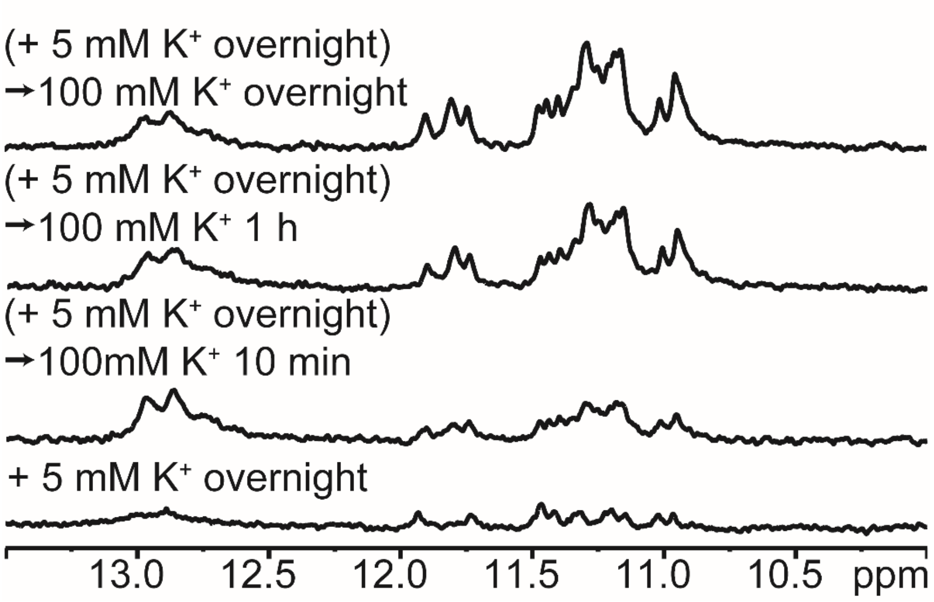

Figure S6. The effect of K^+ concentration on mt10248 G4 structures. The imino proton NMR spectra of mt10248 in 5 mM K^+ solution overnight and after 10 min, 1 h, and overnight addition of K^+ to 100 mM K^+ solution.

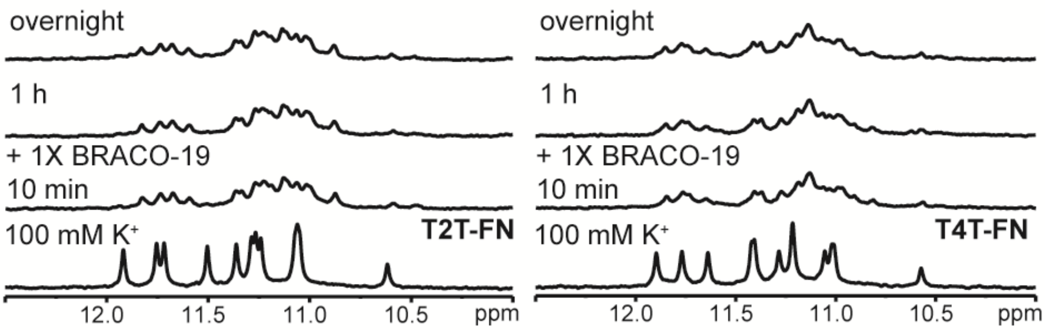

Figure S7. Ligand binding ofBRACO-19 to $(G_3TG_3N_mG_3TG_3)$-FN G4 structures. Imino proton NMR spectra of T2T-FN and T4T-FN in 100 mM K^+ solution and after 10 min, 1 h, and overnight addition of 1 eq. BRACO-19.

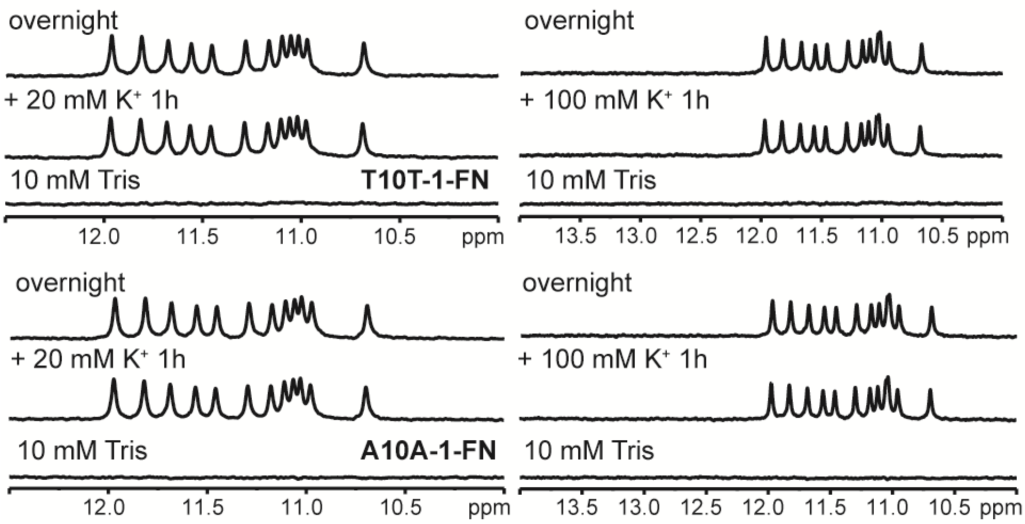

Figure S8. The effect of loop base on G4 formation of $(G_3HG_3N_{10}G_3HG_3)$-FN sequences. NMR spectra of A10A-1-FN and T10T-1-FN in 10 mM Tris and after 1h and overnight addition of 20 mM K^+ and 100 mM K^+.
